# The History and Capabilities of Herbal Simples

**Published:** 1891-06-13

**Authors:** W. T. Fernie


### The History and Capabilities of Herbal Simples.

By W. T. Fernie, M.D.

### XXXII.—STRAWBERRIES.

"Tell me—in mercy tell me"—says Box, the printer, in the immortal farce, "have you a strawberry mark on your left arm?" "No," replies Cox, the hatter. "Then you are he—my long-lost brother," replies Box, overjoyed, and they rush into each other's arms. In like manner it may well be asked of the delicious fruit now under our notice, "Are you a berry? Have you any concern with straw?" "No," would be the correct reply. "Nevertheless, a strawberry you are, and shall be to the end of the chapter." For, in fact, the edible succulent pulp of this favourite fruit is not a berry, but only a juicy cushion over which the numerous small seeds are plentifully dotted, and the name it bears is really a corruption of "strayberry," in allusion to the trailing runners which stray in all directions from the parent stock. So Latimer, in his day, called the non-resident country clergy "strawberry preachers," because they strayed from their parishes, to which they returned only once a year. It is true that a practice obtained of old in many villages to thread these so-called berries on a straw, and then offer them for sale; likewise a custom has been of long standing with gardeners, and is still commonly practised, of putting straw beneath the fruit when beginning to ripen, with the idea of keeping it moist and clean. But there can be no doubt the strayberry plant took its title long prior to these misapprehensions of its nominal purport. In a poem called "London Lyckpenny," written by John Lydgate, about 1480, he says:

"Then unto London I did me hye,
Of all the lands it beareth the pryse;
'Gode Pescode,' one began to cry,
'Strabery ripe,' and 'Cherrys in the ryse.'"

Being in reality of very ancient date, the strawberry is found widely diffused throughout most parts of the world. Among the Greeks its name—Komaros—indicated the compact size of the fruit, which formed scarcely a mouthful. By the Latins it was termed Fragaria, in token of its delicate perfume. Virgil ranked it with sweet smelling flowers; and Ovid gave it a tender epithet, which our gratified palates willingly confirm. Pliny mentions the strawberry as one of the native fruits of Italy. Linnæus declared he kept himself free from gout by eating plentifully of this fruit. And Hoffman says he has known consumption cured by the same means.

From a passage in Shakespeare's play of "Richard the Third," we learn that the esculent was grown at that time in what is now the very heart of London. Gloster, when contemplating the death of Hastings, wishes to get the Bishop of Ely temporarily out of the way, and thus addresses him:—

"My Lord of Ely, when I was last in Holborn,
I saw good strawberries in your garden there;
I do beseech you send for some of them."

Whilst the Elizabethan epicure prized the finer kinds of the cultivated strawberry, doctors then employed the dried leaves as tea, and likewise the roots of the plant as an astringent and remedial medicine. But Dr. Cogan of that era insisted that though sometimes beneficial to the digestion, strawberries were hurtful when taken in considerable quantities with clouted cream; "wherefore," says he, "they that go from Oxford to Botley, or from London to Islington, to eat strawberries and cream, do make but a sleeveless errand." Contrariwise, Fuller taught, "This is a toothsome fruit if taken with claret wine or sweet cream; and," he added, "it is equally salubrious and refreshing when eaten wild."

All former herbalists agreed in pronouncing strawberries as wholesome and beneficial beyond every other English fruit; their smell is refreshing to the spirits, they abate fever, promote urine, and are gently laxative. The leaves may be used in gargles for quinsies and sore mouths; but if anyone suffering from a wound in the head should partake of the fruit, this would certainly prove fatal, in accordance with a widespread superstition.

The chemical constituents of the strawberry are a peculiar volatile aroma, sugar, mucilage, pectine, citric and malic acids in equal parts, woody fibre, and water. The fruit is mucilaginous, somewhat tart, and saccharine. It stimulates perspiration, and imparts a violet scent to the urine. It is especially suitable in inflammatory and putrid fevers, and for catarrhal sore throats.

So wholesome are strawberries that if laid in a heap and left by themselves to decompose, they will decay without undergoing any acetous fermentation; nor can their kindly temperature be soured even by exposure to the acids of the stomach. They are constituted entirely of soluble matter, and leave no residuum to hinder digestion. It is probably for this reason, and because the fruit contains no actual nutriment as food, that the custom arose of combining rich clotted cream with it at table; whilst at the same time the sharp juices are thus agreeably modified.

In Germany stewed strawberries and strawberry jam are taken at dinner with roasted meats or with chicken. If fermented by art the fruit yields an ardent spirit, and a pleasant British wine can be brewed therefrom.

Parkinson advised that water distilled from strawberr ie is good for perturbation of the spirits, and maketh the hear merry. The fruit specially suits persons of a sanguine or bilious temperament, being a surprising remedy for the jaundice of children, and particularly helping the liver of pot-companions, whetters, and drammers. Some also do use thereof to make a water for hot inflammations in the eyes, and to take away any film that beginneth to grow over them. Into a closed glass vessel they put so many strawberries as they think meet for their purpose, and let this be set in a bed of hot horse-dung for twelve or fourteen days, being afterwards distilled carefully and the water kept for use.

French herbalists direct that fresh strawberries recently crushed shall be applied on the face at night for heat spots and freckles by the sun. From the juice, with lemons, sugar, and water, they make a most agreeable drink called Bavaroise à la Grecque. Also they employ the roots and leaves against passive hemorrhages and in chronic diarrhœa.

Properly the strawberry plant is a native of cold climates; and so hardy is it that it bears fruit freely even in Lapland. This fruit, when mixed with reindeer cream and dried in the form of a sausage, makes Kappatialmas, the plum-pudding of the Polar regions.

Strawberry leaves figure on the royal crown of Spain, and on the Imperial diadem of Germany. Eight of these conventional leaves, wrought in gold of a highly ornamental character, form part of the gorgeous coronet which our English dukes claim amongst their privileged insignia, and which they assume on occasions of state ceremonial. Henry the Fourth originated this idea, and introduced the leaves into the royal crown—being determined to add some fresh splendour at the coronation of a Lancastrian prince.

German legends dedicate strawberries to the Virgin, with whom they were a favoured fruit. She went a-berrying with the children on St. John's morning, and therefore on that day no mother who has lost a little child will taste a strawberry, lest her little one should get none in Paradise.

It is to be noticed that though most commonly wholesome and refreshing, yet with some persons, particularly those of a strumous bodily habit, the strawberry always disagrees. The late Dr. Armstrong held a very strong opinion that the seed-grains which lie sprinkled all over the outer surface of each pulpy berry are prone to excite much intestinal irritation; and he advised his patients to suck strawberries through muslin in order to prevent these diminutive seeds from being swallowed.

The fruit of the wild woodland strawberry (*Fragaria vesca*) is more acid than the cultivated strawberry, and is a most excellent cleanser of the teeth, the acid juice dissolving incrustations of tartar without injuring the enamel. If the root of this little plant, which grows abundantly in our shady woods, be held in the mouth, it will effectually stop any flux of blood from the nostrils.

Chemists now make a medicinal tincture from the berries of this woodland strawberry, and find it of curative service for nettlerash and allied erysipelas; likewise for suffocative swelling of the throat and mouth. These brilliant and deliciously sweet little groundlings lie hidden, as Ovid described of old, beneath their modest foliage, whilst the scented air reveals their presence. But a surfeit even of such tempting fruit—the *fraga nascentia humi*—growing wild in the woods, may provoke a painful colic. "*Fugite hinc*," says the poet, "*qui legitis flores!*" "*Frigidus latet anguis in herbâ!*"—"Fly hence before eating to excess. Perchance a cold snake may lurk concealed in the fragrant herbage!"

